# Identifying Genetic Lesions in Ocular Adnexal Extranodal Marginal Zone Lymphomas of the MALT Subtype by Whole Genome, Whole Exome and Targeted Sequencing

**DOI:** 10.3390/cancers12040986

**Published:** 2020-04-17

**Authors:** Patricia Johansson, Ludger Klein-Hitpass, Bettina Budeus, Matthias Kuhn, Chris Lauber, Michael Seifert, Ingo Roeder, Roman Pförtner, Martin Stuschke, Ulrich Dührsen, Anja Eckstein, Jan Dürig, Ralf Küppers

**Affiliations:** 1 Department of Hematology, University Hospital Essen, University of Duisburg-Essen, 45147 Essen, Germany; ulrich.duehrsen@uk-essen.de (U.D.); jan.duerig@sjk.uk-essen.de (J.D.); 2 Institute of Cell Biology (Cancer Research), Medical Faculty, University of Duisburg-Essen, 45147 Essen, Germany; ludger.klein-hitpass@uk-essen.de (L.K.-H.); bettina.budeus@uni-due.de (B.B.); ralf.kueppers@uk-essen.de (R.K.); 3 Institute for Medical Informatics and Biometry, Faculty of Medicine Carl Gustav Carus, Technical University Dresden, 01307 Dresden, Germany; matthias.kuhn@tu-dresden.de (M.K.); chris.lauber@tu-dresden.de (C.L.); michael.seifert@tu-dresden.de (M.S.); ingo.roeder@tu-dresden.de (I.R.); 4 Department of Oral and Cranio-Maxillofacial Surgery, Kliniken Essen-Mitte, Evang. Huyssens-Stiftung/Knappschaft GmbH, University Hospital of Essen, 45136 Essen, Germany; r.pfoertner@kliniken-essen-mitte.de; 5 Department of Radiotherapy, University Hospital Essen, 45147 Essen, Germany; martin.stuschke@uk-essen.de; 6 Department of Ophthalmology, Molecular Ophthalmology Group, University of Duisburg-Essen, 45147 Essen, Germany; anja.eckstein@uk-essen.de; 7 German Cancer Consortium (DKTK), 45147 Essen, Germany

**Keywords:** ocular adnexal lymphoma, JAK/STAT, NOTCH, pathogenesis, somatic mutation

## Abstract

The pathogenesis of ocular adnexal marginal zone lymphomas of mucosa-associated lymphatic tissue-type (OAML) is not fully understood. We performed whole genome sequencing (WGS) and/or whole exome sequencing (WES) for 13 cases of OAML and sequenced 38 genes selected from this analysis in a large cohort of 82 OAML. Besides confirmation of frequent mutations in the genes transducin beta like 1 X-linked receptor 1 (*TBL1XR1*) and cAMP response element binding protein (*CREBBP*), we newly identifed *JAK3* as a frequently mutated gene in OAML (11% of cases). In our retrospective cohort, *JAK3* mutant cases had a shorter progression-free survival compared with unmutated cases. Other newly identified genes recurrently mutated in 5–10% of cases included members of the collagen family (collagen type XII alpha 1/2 (*COL12A1*, *COL1A2*)) and *DOCK8*. Evaluation of the WGS data of six OAML did not reveal translocations or a current infection of the lymphoma cells by viruses. Evaluation of the WGS data for copy number aberrations confirmed frequent loss of *TNFAIP3*, and revealed recurrent gains of the NOTCH target *HES4*, and of members of the CEBP transcription factor family. Overall, we identified several novel genes recurrently affected by point mutations or copy number alterations, but our study also indicated that the landscape of frequently (>10% of cases) mutated protein-coding genes in OAML is now largely known.

## 1. Introduction

Lymphomas of the ocular adnexa are a rare entity, accounting for about 1–2% of non-Hodgkin lymphomas (NHL) [1]. In about 80% of cases, extranodal marginal zone lymphomas of the mucosa-associated lymphatic tissue (MALT) subtype are the most frequent type of primary ocular adnexal lymphomas [2,3,4]. Ocular adnexal MALT lymphomas (OAML) have a mature B cell phenotype and derive from post-germinal center B cells [5,6]. In some geographical areas, chronic infections by *Chlamydia psittaci* have been linked to the pathogenesis of OAML [7,8]. Chromosomal translocations that are frequent in particular subtypes of extranodal marginal zone lymphomas—t(1;14)(p22;q32) (*BCL10*/IgH), t(14;18)(q32;p21) (IgH/*MALT1*), t(11;18)(q21;q21) (*BIRC3*/*MALT1*), and t(3;14)(p14;q32) (*FOXP1*/IgH)—have also been identified in OAML, but their overall frequency in OAML is low [9,10,11,12]. As these translocations promote constitutive NF-κB activity [9,10,12], which is a hallmark of MALT lymphomas [13], several studies searched for further mutations in components of the NF-κB pathway in OAML. Indeed, recurrent mutations were found in *TNFAIP3* (around 30% of cases), *MYD88* (around 5–20% of cases), and *BCL10* (around 25% of cases in a cohort of patients from China), whereas other genes were rarely or not mutated in the cases analyzed [14,15]. We recently showed that also *NFKBIA* is recurrently mutated in such lymphomas [16]. Only 6% of patients in our cohort harbored *BCL10* mutations, and *TNIP1* was mutated in 5% of cases. Moreover, we identified *NOTCH1*, *NOTCH2*, and *KMT2D*, encoding an epigenetic regulator, as further recurrently mutated genes in OAML [16]. Another candidate gene analysis revealed recurrent mutations in *TBL1XR1* (transducin beta like 1 X-linked receptor 1; 6% of cases), *TNFRSF14*, (tumor necrosis factor receptor family member 14; 5%), and *TET2* (tet methycytosine dioxygenase 2; 4% of cases), besides a few less frequently mutated genes [17]. A further candidate gene mutation analysis validated several genes identified by the other studies, mainly adding *KMT2C* to the list of genes recurrently mutated in OAML [18].

Thus far, only one genome-wide mutation study of OAML has been published [19]. This whole genome sequencing (WGS) analysis included 10 cases. Additional cases were then investigated by targeted sequencing. Besides validation of mutations in NF-κB pathway members, and in *TBL1XR1*, *KMT2D*, and *NOTCH1*, this analysis newly identified mutations in cAMP response element binding protein (*CREBBP;* 17% of OAML in the extended cohort), encoding a histone/protein acetyltransferase, and in *LRP1B* (6% of cases).

In the present work, we performed WGS for six cases of OAML, and whole exome sequencing (WES) for eight cases, including one case studied by both approaches. On the basis of the results from these studies, 38 mutated genes were selected for a target gene panel, and samples from 82 OAML were studied by targeted deep sequencing for mutations in these genes. Finally, we also investigated the WGS data for translocations, gains and losses, and the presence of viral DNA.

## 2. Results

### 2.1. Whole Exome, Whole Genome, and Targeted Sequencing of OAML

We analyzed primary OAML from five patients for somatic mutations by WGS, from seven other patients by WES, and one further OAML by both sequencing approaches. Clinical data of the cohort and detailed characteristics of the patients are shown in Table 1 and Table S1. The WES analysis was revealed after filtering 139 heterozygous non-synonymous mutations in 138 genes (Table S3). Known SNPs were excluded from the analysis. The mean number of mutations per patient was 17.6 (range 1-41). The WGS analysis was revealed after filtering eight recurrently mutated genes in six patients, which carried the “impact moderate and high”, as described above (Table S2). The low number of mutations was explained by the stringent filtering criteria applied to this sequencing.

We selected 38 genes for a targeted sequencing analysis of a large collection of OAML from 82 patients, including 11/13 index cases (Table S2). The selected genes included 36 genes mutated in at least one of the six OAML studied by WGS and/or at least in two of the eight lymphomas studied by WES, as well as two additional genes (*CXCR4*, *ACTN4*). Mutations in at least 1/38 genes analyzed by targeted sequencing were observed in 50/82 cases (61%), and 32 (39%) patients exhibited no mutations (Table S2). The internal controls used for targeted sequencing from different tissue types of the same sample revealed an overlap between paired samples of 95%, independent of tissue type. Only one of the samples per patient was therefore considered in the further analysis. A sample from a reactive hyperplasia revealed no mutations. The mean number of mutations per patient was 1.33 (range 0–15). Variant allele frequencies were between 10% and 72%, reflecting homo- and heterozygous mutations.

### 2.2. Detection of Numerous Recurrently Mutated Genes in OAML

**Figure 1:**
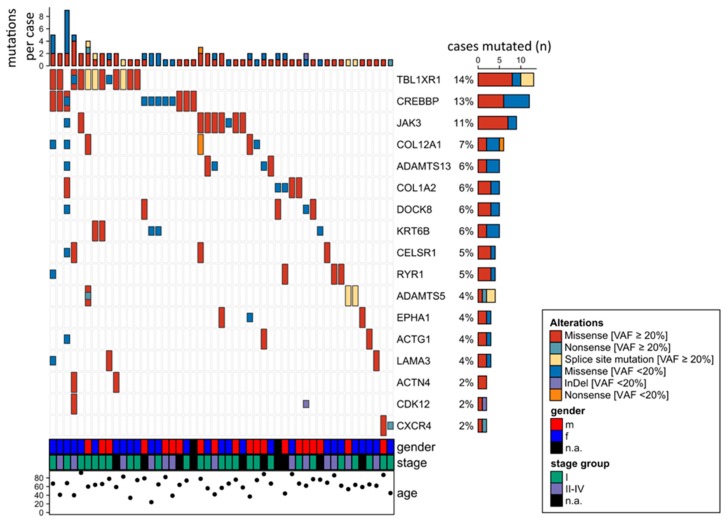
Overview of 17 genes mutated in at least two cases in the ocular adnexal marginal zone lymphomas of mucosa-associated lymphatic tissue-type (OAML) cohort analyzed by targeted amplicon sequencing of 38 genes. Mutations observed in whole genome sequencing (WGS) or whole exome sequencing (WES), but not verified by targeted deep sequencing were not taken into account, considering the higher quality and sequencing depth of the latter approach. For the same reasons, mutations seen by targeted sequencing but not covered or filtered out in the WES or WGS analyses are included in this overview. Cases 101 and 102 are not considered here, because only WES and/or WGS were performed for those cases, but not targeted sequencing.

Figure 1 gives an overview on the 17 genes mutated in at least two of the 82 OAML analyzed by targeted sequencing. The most frequently mutated gene (14% of cases) was *TBL1XR1*, an F-box-like/WD (tryptophan-aspartic acid) repeat-containing protein that is a component of both nuclear receptor corepressor (N-CoR) and histone deacetylase 3 (HDAC 3) complexes. It is required for transcriptional activation by a variety of transcription factors [20]. *TBL1XR1* mutations were mostly detected with variant allele frequencies ≥ 20%, indicating a clonal representation (Figure 1).

The second most frequently mutated gene was cAMP response element binding protein (*CREBBP*), with 13% mutated cases. CREBBP belongs to the KAT3 family of histone/protein lysine acetyltransferases and is a coactivator of many transcription factors.

**Figure 2:**
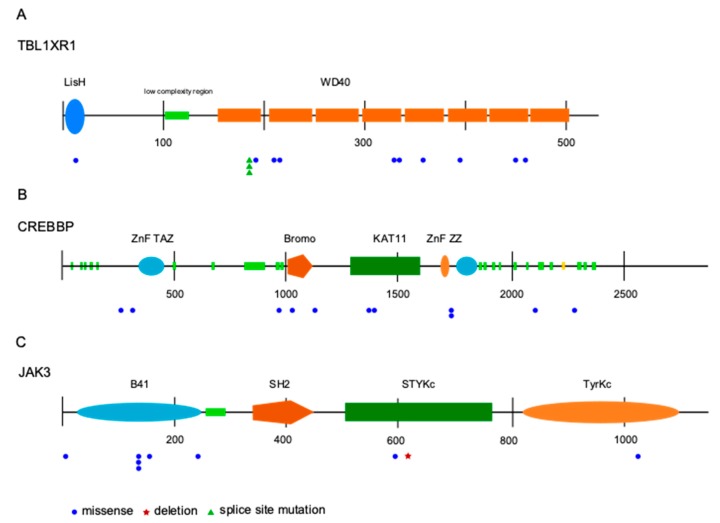
Distribution and type of mutations in the three most recurrently mutated genes identified by targeted sequencing, transducin beta like 1 X-linked receptor 1 (*TBL1XR1*) (**A**), cAMP response element binding protein (*CREBBP*) (**B**), and *JAK3* (**C**). The coding region on mRNA level is shown. Numbers indicate basepairs. Abbreviations: LisH—lis homology domain; WD40—beta-transducin repeats; ZnF—zinc finger domain; TAZ—transcription adaptor putative zinc finger; Bromo—Bromo domain; KAT11—histone acetylation protein domain; ZZ—ZZ-type zinc finger domain; B41—band 4.1 homologues; SH2—Src homology 2 domain; STYKc—protein kinase, unclassified specificity; TyrKc—tyrosine kinase, catalytic domain.

**Figure 3:**
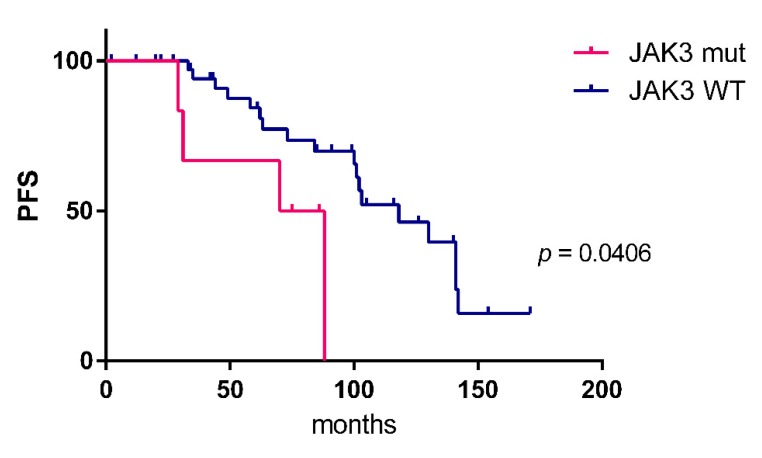
Kaplan–Meier estimates (progression-free survival, PFS) of patients with (*n* = 6) and without *JAK3* mutation (*n* = 39). The Log-rank (Mantel–Cox) test was applied. No differences in overall survival were observed.

*JAK3* was identified as the third most frequently mutated gene. Mutations were observed in 9/82 patients (11%), two samples harbored subclonal mutations with a variant allele frequency below 20%. All mutations were missense mutations, mostly located in the FERM domain. Other mutations were distributed in the pseudokinase and kinase domain (Figure 2). Analyzing the survival data in this patient cohort, we observed that *JAK3* mutant cases had a shorter progression free survival (PFS) compared to unmutated cases (Figure 3). No differences in overall survival were observed. For the two other most frequently mutated genes, TBL1XR1 and CREBBP, no significant association of the presence of mutations with progression-free or overall survival was obtained.

### 2.3. Analysis of WGS Data for CNVs, Chromosomal Translocations, and Viral Sequences

**Figure 4:**
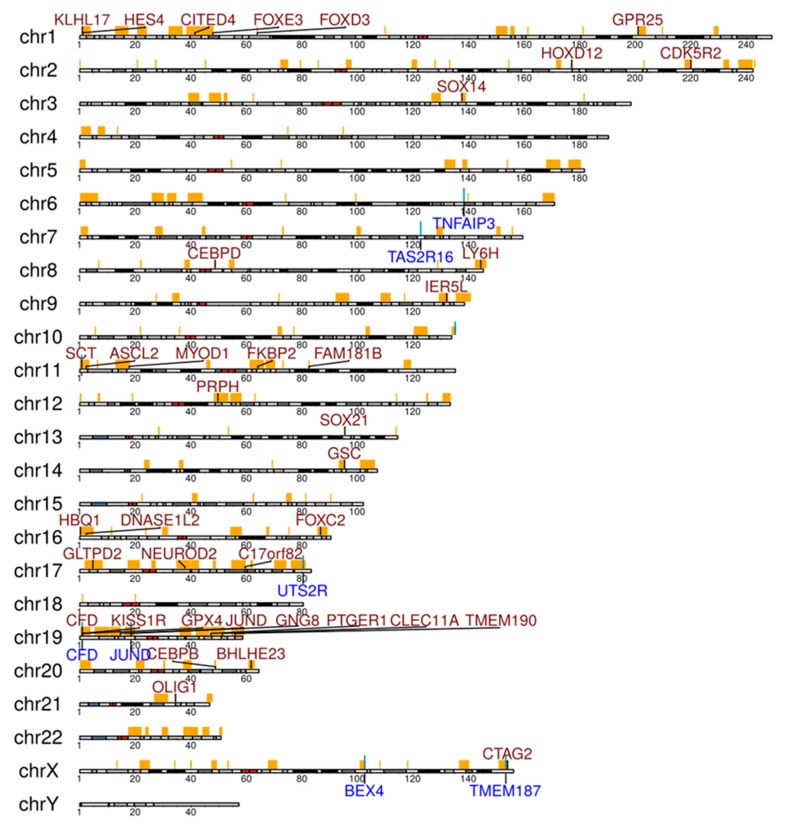
Selected genes in significantly enriched regions of gains (genes written in red) and losses (genes written in blue) analyzed by copy number analysis from the WGS analysis of six OAML. Regions with false discovery rate (FDR)-corrected *p*-values below 0.05 from Fisher’s exact tests were considered as being significantly enriched in patients. Regions with gains or losses in at least one patient are marked at the respective chromosome in orange.

Somatic CNV calling revealed 167 loss and 3437 gain regions (overview displayed in Figure S1). Most frequent losses were observed on chromosome 6 at the locus 6q23.3, including the *TNFAIP3* gene in three of six patients (Table S4). Numerous genes showed recurrent gains in the six cases (Figure 4, Table S4). Although not all gains in a cancer have pathogenetic roles, several genes with recurrent gains in our collection deserve mentioning. *HES4* showed gains in four of the cases. HES4 is a downstream target and effector of NOTCH activity [21]. *CEBPB*, which showed gains in four OAML, functions as transcription factor in myeloid cells, but it also has a pathogenetic role in multiple myelomas, and positively regulates BCL2 in B cells [22,23]. *CEBPD*, encoding another member of the CEBP family, was also recurrently affected by gains (4/6 cases). Little is known about the role of CEBPD in B cells, but it is mutated in a Burkitt lymphoma cell line, pointing to a potential role in B cell malignancies [24].

In the translocation analysis, none of the translocations described in MALT lymphomas (i.e., t(1;14) (p22;q32) (*BCL10*/IgH), t(14;18)(q32;p21) (IgH/*MALT1*), t(11;18)(q21;q21) (*BIRC3*/*MALT1*), and t(3;14)(p14;q32) (*FOXP1*/IgH)) were observed in the WGS data. We also did not find any convincing indication of other novel translocation events.

In a search for viral sequences, no conclusive hits against eukaryotic viruses were obtained.

### 2.4. Analysis for Bacterial Sequences in the OAML Tissues

Because many MZL subtypes develop by triggering certain bacteria, such as *Helicobacter pylori* in gastric MALT or *Chlamydophila psittaci* in OAML (at least in some geographical regions), we performed a metagenomic analysis of bacterial DNA in 17 samples. We also did not identify DNA of *C. psittaci* in any of the six lymphomas studied by WGS (data not shown). No other bacteria were recurrently detected in these lymphomas.

## 3. Discussion

Previous studies on somatic mutations and chromosomal abnormalities in OAML mostly focused on candidate gene approaches, interrogating genetic lesions that were known from other types of B cell lymphomas [10,11,14,15,16,25,26,27]. This led to major insights into recurrent gene mutations, chromosomal translocations, and copy number gains and losses in OAML. A major finding was that somatic mutations affecting genes of the NF-κB pathway are a hallmark of OAML, but that translocations deregulating NF-κB pathway members are relatively rare in comparison to other MALT lymphomas [9,10,11,12,28]. However, the question remained as to whether the landscape of frequently mutated genes in OAML is now known, or whether numerous other main drivers have not been detected in the prior focused mutation studies. We therefore followed the strategy to first perform unbiased WGS and/or WES of 13 cases of OAML and then select candidate genes from these genome-wide mutation screens to study a large cohort of cases for recurrence of these mutations. We selected 38 candidate genes and studied them for mutations in 82 OAML by deep sequencing. Furthermore, the WGS data were also analyzed for chromosomal translocations, CNVs, and viral sequences.

The absence of translocations affecting the genes *BCL10*, *MALT1*, *BIRC3*, or *FOXP1,* as they are frequently seen in other types of MALT lymphomas, in six OAML analyzed by WGS is in line with published data [29]. We also did not detect other novel translocations. Thus, chromosomal translocations apparently have only a minor role in the pathogenesis of OAML.

The presence of *Chlamydophila psittaci* in OAML as a potential bacterial trigger for this lymphoma is described only in some geographical areas [7,8]. We performed a bacterial metagenomic analysis of 17 OAML and did not identify *C. psittaci* in our cohort. Other bacteria were not recurrently detected in that analysis either. Furthermore, we analyzed the WGS data of six OAML for the occurrence of pathogenic viruses infecting humans, but did not observe any viral sequences. Hence, neither bacteria nor known viruses appear to play a substantial or any role in the pathogenesis of OAML, at least in patients from Germany.

The copy number variation analyses performed on WGS data revealed overall and recurrently more gains than losses in this cohort (Table S4). Among genes in areas with losses, *TNFAIP3* as a major inhibitor of the NF-κB signaling pathway is affected by chromosomal copy number loss, validating prior studies [10,13,26,27,30]. Among genes with recurrent gains in our cohort, HES4 and two members of the CEBP transcription factor family may be particularly of interest. The gains in *HES4* fit to the indications that NOTCH pathway activation is important in OAML. We previously reported recurrent activating mutations of *NOTCH1* and *NOTCH2* in OAML [22,23]. Thus, copy number gains of *HES4* may be a further mechanism for enforced NOTCH pathway activation in OAML. Although CEBP family members are mainly known for their function in myeloid cells, there are indications that CEBPB and CEBPD may have roles in B lineage malignancies [21,22,23].

Regarding the mutational analysis of 38 candidate genes selected from the WES and WGS studies in 82 cases of OAML, 9 of the 38 genes were not mutated, which is explained by applying different filtering criteria for targeted sequencing compared to filtering criteria applied for WGS and WES due to different pipelines for analysis. Moreover, the tumor cell content in formalin-fixed paraffin-embedded (FFPE) material used for targeted sequencing was lower compared to the tumor cell content of sorted tumor cells for WGS and WES, resulting in a lower variant allele frequency in targeted sequencing. Additionally, tumor-derived mutations were compared against non-tumor mutations in WGS and WES. In targeted sequencing and WES, the mutations were filtered for variant allele frequency and quality score; in WGS, the mutations were required to occur in both read directions. We focus in the following discussion on those of the 38 genes that were mutated in at least two of the cases (Figure 1). The most frequently mutated gene was *TBL1XR1*, with mutations in 14% of the cases, validating two prior studies that detected frequent *TBL1XR1* mutations in OAML [17,19]. This gene can activate the transcription of transcription factors such as NF-κB and JUN, and hence may contribute to the strong NF-κB activity in OAML. *TBL1XR1* is mutated in various tumors, promoting tumor cell survival, and has been linked to a poor prognosis [31,32]. In one study, *TBL1XR1* mutant cells exhibited a 10–15% higher proliferation rate than wild-type *TBL1XR1* cells [19], suggesting a survival advantage for lymphoma cells carrying this mutation.

The second most frequently mutated gene in our cohort was *CREBBP* (13% of cases). Thus far, only one prior study had detected *CREBBP* mutations in OAML [19]. We therefore provided a valuable validation of frequent mutations in this gene in OAML. CREBBP, which is also mutated in other types of B cell lymphomas [33], is an epigenetic regulator, belonging to the KAT3 family of histone/protein lysine acetyltransferases. Mutations were also reported in other studies for the epigenetic regulators *KMT2D* and *KMT2C* [16,18]. Therefore, epigenetic dysregulation seems to be involved in the pathogenesis of OAML.

The JAK/STAT pathway has not yet been reported as playing a role in the pathogenesis of OAML. We here identified for the first time *JAK3* mutations in OAML. With 11% of patients harbouring *JAK3* mutations, it is the third most frequently mutated gene in our cohort. All mutations were located at positions known to alter the protein function (Figure 2) and leading to activation of the JAK/STAT signaling pathway by causing a gain of function of JAK3 [34,35,36]. Analyzing the survival data in this cohort, we observed that *JAK3* mutant cases have a shorter PFS compared to unmutated cases (Figure 3). Because OAML have a high relapse rate of up to 50% after several years including systemic relapses [37,38], the information on mutations leading to a shorter PFS could be used to modify initial treatment and/or monitoring these patients more closely. Identification of activating JAK3 mutations in OAML also represent a possible therapeutic approach by targeting the JAK/STAT pathway with JAK inhibitors [39]. However, as this is a retrospective analysis, validation of the link of *JAK3* mutation to clinical behaviour needs to be validated in future studies.

Further recurrently mutated genes include *COL12A1*, collagen type XII alpha 1 chain, which plays a role in cell adhesion. Different splicing variants occur in colorectal carcinoma [40], and mutations are reported for resistant small-cell lung cancers [41] and papillary renal cell carcinoma [42]. Notably, recurrent deletions of *COL12A1* have been identified in primary central nervous system lymphoma [43], and thus we now have identified a second B cell lymphoma with recurrent genetic alterations of this gene. The gene of a second collagen family member, COL1A2, was mutated in 6% of cases. Although no mutations of *COL1A2* have previously been reported for lymphomas, a potential pathogenetic role of these mutations is indicated from the finding that it is frequently epigenetically silenced in medulloblastoma and colorectal cancer [44,45]. A further recurrently mutated gene in OAML (6% of cases) is *DOCK8*. The fact that DOCK8 is involved in RhoGTPase signaling, that germline loss of *DOCK8* causes an immunodeficiency, that it has an important role in murine B cell differentiation, and that mutations of this gene have previously been reported in other cancers [46,47], point to a potential involvement of *DOCK8* mutations in the pathogenesis of OAML.

## 4. Materials and Methods 

### 4.1. Patients and Samples

Patient samples were obtained from fresh and FFPE material of patients treated at the University Hospital Essen and the Kliniken Essen-Mitte. A total of 84 samples were selected for analysis with approval of the ethical review committee of the University of Duisburg-Essen. This research was approved by the Ethics Committee of the Medical Faculty of the University Duisburg-Essen on 04.12.2012 (ethic code: 12-5001-BO). Clinical data of these patients are summarized in Table 1. Samples used for sequencing analyses were collected from untreated patients. Patients were diagnosed between 2005 and 2016 in accordance with the WHO 2016 classification [48]. Only patients with primary OAML were included in this study. Samples obtained from fresh biopsies were either immediately processed or suspensions were prepared and cryopreserved. Samples were only further analyzed if the diagnosis of OAML was verified. For diagnosis, the corresponding immunohistochemical stainings were evaluated on FFPE sections with antibodies against CD3, CD5, CD10, CD20, CD23, CD79a, CXCR4, immunoglobulin light chains, cyclin D1, and Ki-67 (details not shown). For cases in which monoclonality could not be determined by light chain restriction, the diagnosis was complemented by flow cytometry analysis of the respective cell suspension and/or a molecular analysis for clonal immunoglobulin heavy chain gene rearrangements. WGS was performed in six cases with paired tumor and non-tumor samples. WES was performed for eight cases with paired tumor and non-tumor samples available (Table S1). 

### 4.2. Tumor and Non-Tumor Cell Enrichment

For WGS analyses, tumor cells were enriched by fluorescence-activated cell sorting (FACS; Aria III, BD Biosciences; Heidelberg, Germany) after staining with antibodies against CD19, and immunoglobulin kappa and lambda light chains (BD Biosciences). For cases without light chain restriction, cells were sorted for positivity of CD19, CD27, and IgD (BD Biosciences). To obtain non-tumor controls, we performed density gradient centrifugation of heparinized peripheral blood samples of the respective patients (Pancoll human, PAN-biotech, Aidenbach, Germany). Peripheral blood mononuclear cells (PBMCs) depleted for CD19^+^ B cells by magnetic cell separation with a bead-coupled anti-CD19 antibody (MACS, Miltenyi Biotech, Bergisch Gladbach, Germany) served as non-tumor controls. For WES, tumor cells were obtained from fresh biopsies, as described above. As non-tumor controls, T cells were enriched from PBMCs by MACS with an anti-CD3 antibody. After tumor cell enrichment, the purity of all samples for WGS and WES was at least 95%, as determined by flow cytometry.

### 4.3. DNA Isolation

DNA was extracted from the isolated cells with the QIAamp Blood mini kit (Qiagen, Hilden, Germany). For FFPE material DNA was extracted from up to eight 10 µm thick sections per sample using the QIAamp DNA FFPE Tissue Kit (Qiagen, Hilden, Germany). DNA concentrations were measured with a Qubit Fluorometer 2.0 (Life Technologies, Darmstadt, Germany). Between 175 ng and 74 µg genomic DNA was isolated from the samples. 

### 4.4. Whole Genome Sequencing

WGS was performed for six cases with paired tumor cells and non-B cell PBMC samples. Genomic DNA was sheared to a size of 300 bp with a Covaris S220 ultrasonicator (Covaris Inc., Woburn, MA, USA). Libraries were prepared with NexteraXT DNA sample preparation kit with 1 ng DNA. Whole genome paired-end sequencing was carried out on a HiSeq 2500 apparatus (Illumina, San Diego, CA, USA). Sequencing reads were trimmed on the basis of quality and adapter sequences with the tool Trim Galore! (Babraham Bioinformatics, Babraham Institute, Cambridge, U.K.). Reads passing quality control were mapped to human reference genome hg38 with BWA [49]. The average sequence coverage was 21 (range 6 to 59). The SNV-caller Platypus [50] generated a list of putative SNVs on the basis of the mapping files, and we added annotations with SnpEff [51]. The annotated list was further shortened as follows: SNVs predicted with impact less than moderate by SnpEff were filtered out, reads with low coverage for the variant (threshold: < 5 variant reads) were removed, the variant must occur in at least two forward and two reverse reads, no significant strand bias was allowed to be detected, variants must occur in at least one of the tumor samples and not in the non-tumor samples, and variants annotated in dbSNP (v132) were removed. Genes affected by SNVs were analyzed for an enrichment of known cancer signaling pathways. Pathway annotations were taken from ConsensusPathDB [52]. Numbers of affected genes per pathway were counted and pathway enrichment was determined by Fisher’s exact test. Data are available under accession number xxx (*to be provided later*) in the NCBI Sequence Read Archive.

### 4.5. Gene Copy Number Analysis

Gene copy number alterations were determined on the basis of WGS data. Sequencing reads were trimmed on the basis of quality and adapter sequences with Trim Galore! (Babraham Bioinformatics, Babraham Institute, Cambridge, UK). Reads that passed quality control were mapped to the human reference genome hg19 with BWA-MEM (https://arxiv.org/abs/1303.3997). Read coverage of protein-coding genes for tumor and normal samples were determined with the Genome Analysis Tool Kit (GATK 3.5) [53] using the tool DepthOfCoverage. Mean coverages were computed for both samples of each tumor-normal pair and used to shift the mean coverage of the sample with the lower coverage to the mean coverage of the sample with the higher coverage by adding the difference of both means to the coverage of each individual gene in the sample with the lower coverage. This ensures that both samples are comparable under the assumption that the majority of genes do not differ in their copy number between tumor and normal samples. Finally, log_2_-ratios of tumor to normal were computed for each gene to obtain a log-ratio profile for each tumor-normal pair. Gains or losses of genes with a false discovery rate (FDR)-corrected *p*-value below 0.05 from Fisher’s exact tests were considered as being significantly enriched in patients.

### 4.6. Search for Viral Sequences

WGS data from six OAML were analyzed for sequences with a detectable degree of sequence homology to known viruses. The sequencing reads were aligned to the human reference genome using BWA [49], and sequencing reads not aligning to the human reference were retained. The number of these unmapped reads ranged from 4–50 × 10^6^ between the tumor samples. In a first search, the unmapped reads were assembled using SPAdes [54], and the resulting contigs were submitted to a BLAST [55] search at the nucleotide level (BLASTN) against a database of viral sequences obtained from the *RINS* package [56]. This viral database contained 32,102 genome sequences of all known viruses. In a second search, the unassembled reads, such as raw sequencing reads, were submitted to a BLAST search at the protein level (tblastx) against the same virus database. 

### 4.7. Translocations Analysis

Translocation analysis was performed on the basis of WGS data that were trimmed and mapped to the human reference genome, as described for the gene copy number analysis. BreakDancer [57] was used to search for trans-chromosomal rearrangements with a minimum alternative mapping quality of 10. The results were reviewed manually for already known translocations occurring in MALT lymphomas.

### 4.8. Whole Exome Sequencing

WES was performed for eight cases with paired tumor and non-tumor T cell samples. A total of 50 ng of DNA was sheared to a size of 300 bp with a S220 ultrasonicator (Covaris). Libraries were prepared from isolated tumor cells and controls with the NEBNext Ultra DNA library prep kit (New England Biolabs, Ipswich, MA, USA). WES was carried out after capturing with NimbleGen SeqCap EZ enrichment kit v3 (Roche NimbleGen, Madison, WI, USA) on the HiSeq 2500 system with 2 × 101 bp paired-end reads (Illumina). More than 90% of the targeted exome had a minimum coverage of 20. For de-multiplexing and adapter trimming CASAVA v1.7 (Illumina) and Trimmomatic [58] were used. Read duplicates were removed with SAMtools rmdup [59]. The alignment was performed with BWA [49] against GRCh37 (hg19). Strand Avadis NGS software (Strand Genomics Inc.: San Francisco, CA, USA) was used for variant calling. Genes affected by SNVs were analyzed for enrichment of known cancer signaling pathways, as described for the WGS analysis.

### 4.9. Determination of the Coding Sequence of Bacterial 16S Ribosomal RNA

For 17 samples, we performed amplicon-based DNA sequencing of the prokaryotic variable regions V3 and V4 of the 16S ribosomal RNA. DNA was extracted from suspensions prepared from whole tissue biopsies using the QIAamp Blood mini kit (Qiagen). A positive control (*Escherichia coli*) and a no-template control were included. A total of 50 ng genomic DNA was used for the PCR, and 25 cycles of PCR were performed as published in the Illumina 16S Library Preparation Workflow. Primers [60] were combined with Illumina adapters (see Illumina 16S Library Preparation Workflow). After magnetic bead-cleanup (Agencourt AMPure XP, Beckman Coulter, Krefeld, Germany), eight further cycles of PCR were performed to add NexteraXT indices (Illumina) for multiplexing, followed by a second clean-up. Sequencing was carried out on an Illumina MiSeq apparatus. 

### 4.10. Targeted Re-Sequencing

Genes for targeted re-sequencing were selected on the basis of the following criteria. In the WES analysis, genes had to be recurrently mutated in at least two patients. A high quality score > 500 was set as determined by Strand Avadis NGS software. In the WGS analysis, genes mutated in at least one patient were selected when the filtering criteria according to SnpEff [51] tool were set to “impact moderate and high”. We further added *CXCR4* to the candidate list, although it was neither mutated in the WGS nor the WES analysis, as its high expression in OAML [61] raised our interest in this gene. *ACTN4* was included in the list of target genes, although it was mutated only in one of the lymphomas studied by WES, as it can function as a co-activator of NF-κB [62].

After selection of 37 mutated genes plus *CXCR4* (Table S2), a custom panel was designed for the coding exons of those 38 genes using the IonApliSeq platform (Thermo Fisher Scientific, Waltham, MA, USA). Sequencing was carried out on an IonProton sequencing apparatus (Thermo Fisher). For 82/84 patients, enough material was available for targeted re-sequencing. A total of 91 samples from those 82 patients were used, including 11/13 index patients (seven from the WES analysis, four from the WGS analysis). For three lymphoma samples, the sequencing quality was not sufficient, and therefore these samples were removed. Six samples were included as additional internal controls, which were one patient exhibiting a reactive lymphoid hyperplasia as precursor lesion with no evidence for a lymphoma, and four patients for whom one or two additional sample types of the same biopsy were available. These were two patients with a paired sample of FFPE and a suspension; one patient with a suspension and sorted tumor cells; and one patient with FFPE material, sorted tumor cells, and a cell suspension. The distinct samples from the same patient were compared to each other and revealed the same mutations in most cases. The samples additionally included for internal control were then excluded from further analyses, and hence only one sample per patient was kept for the final analysis. The samples finally included in the evaluation were 63 FFPE samples, 10 cell suspensions, and 11 samples of sorted tumor cells.

Filtering criteria were set for non-synonymous mutations only and novel SNVs only. Frameshift mutations were manually reviewed and removed, if located in homopolymer stretches or repeats. Mutations with a strand bias ≥ 100 according to Strand Avadis NGS were removed. The variant allele frequency was set to a minimum of 10%.

## 5. Conclusions

We conclude from our WGS, WES, and targeted deep sequencing analysis of the 38 candidate genes that the majority of frequently mutated protein-coding genes involved in the pathogenesis of OAML apparently have been identified, as we detected only one novel gene mutated in more than 10% of the cases (*JAK3*). Importantly, we also identified a substantial number of recurrently mutated genes with frequencies of mutated cases between 2–10%. This means that the landscape of genetic lesions in OAML is quite complex and diverse, and that besides a number of frequently mutated oncogenes and tumor suppressor genes, a large variety of additional genes are mutated. For most of these genes mutated at low frequency, it remains unclear as to whether they are driver genes in this lymphoma, or whether their contribution to the malignant transformation process is minor.

We confirmed previously published data on mutations occurring in OAML, observed no hits in a search for bacterial and viral triggering factors in OAML, and identified involvement of the JAK/STAT signaling pathway in OAML by detecting frequent mutations in *JAK3*, linked to a disadvantage in PFS in these patients. Evaluation of the WGS data for copy number aberrations confirmed frequent loss of *TNFAIP3*, and revealed recurrent gains of the NOTCH target *HES4*, and of members of the CEBP transcription factor family.

## Figures and Tables

**Table 1:** Patients’ characteristics.

Characteristics	No. of Patients (*n* = 84)	Percentage of Patients
Age at diagnosis (years)		
Median	66	
Range	24–92	
Sex		
Male	40/79	51
Female	39/79	49
Ann-Arbor stage ot diagnosis		
IEA	48/66	73
II–IV	18/66	27
Localisation at diagnosis		
Orbita	29/72	40
Conjunctiva	18/72	25
Lid	18/72	25
Lacrimal gland	7/72	10

## Supplementary Materials

The following are available online at https://www.mdpi.com/2072-6694/12/4/986/s1, Figure S1: Gene copy number alteration profiles of six OAML, Table S1: Overview of clinical data and the sequencing analyses performed, Table S2: Mutations identified in the 38 genes analyzed by targeted deep sequencing, Table S3: Significant enrichment of gains and losses in six OAML studied by WGS, Table S4: Significant recurrent enrichment of gains and losses in six OAML studied by WGS.
