# Supplementary material for: Identifying Genetic Lesions in Ocular Adnexal Extranodal Marginal Zone Lymphomas of the MALT Subtype by Whole Genome, Whole Exome and Targeted Sequencing
Source: Cancers (Basel). 2020 Apr 17;12(4):986. doi: 10.3390/cancers12040986 (PMC7225979; doi:10.3390/cancers12040986)
Supplement: Supplementary file 1 [file cancers-12-00986-s001.zip › cancers-756005-SUPP/cancers-756005-supp-final.pdf]

Supplementary Materials: Identifying Genetic Lesions in Ocular Adnexal Extranodal Marginal Zone Lymphomas of the MALT Subtype by Whole Genome, Whole Exome and Targeted Sequencing

Patricia Johansson, Ludger Klein-Hitpass, Bettina Budeus, Matthias Kuhn, Chris Lauber, Michael Seifert, Ingo Roeder, Roman Pförtner, Martin Stuschke, Ulrich Dührsen, Anja Eckstein, Jan Dürig and Ralf Küppers

Case 1

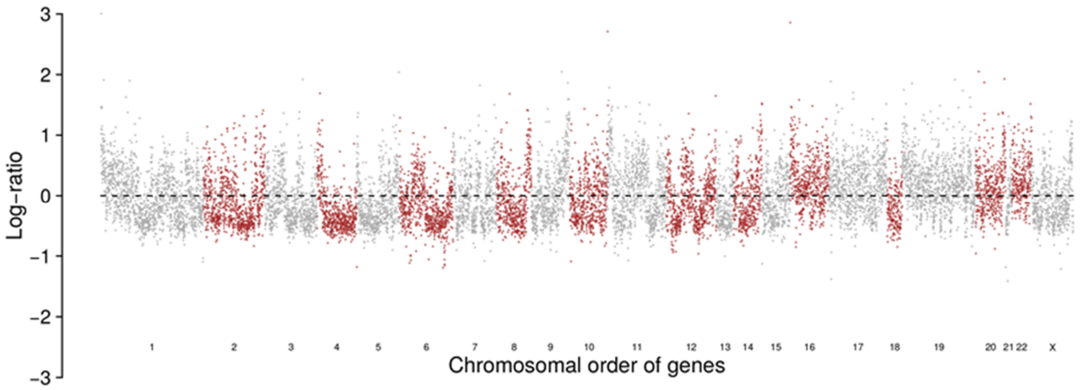

Case 6

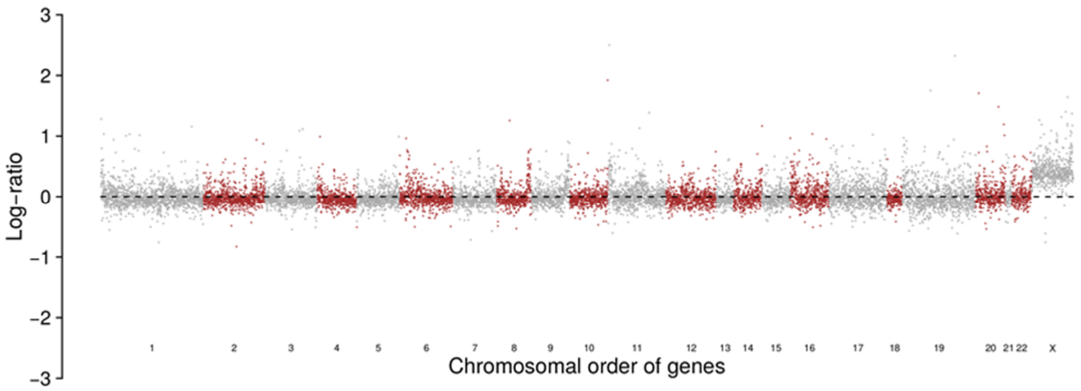

Case 31

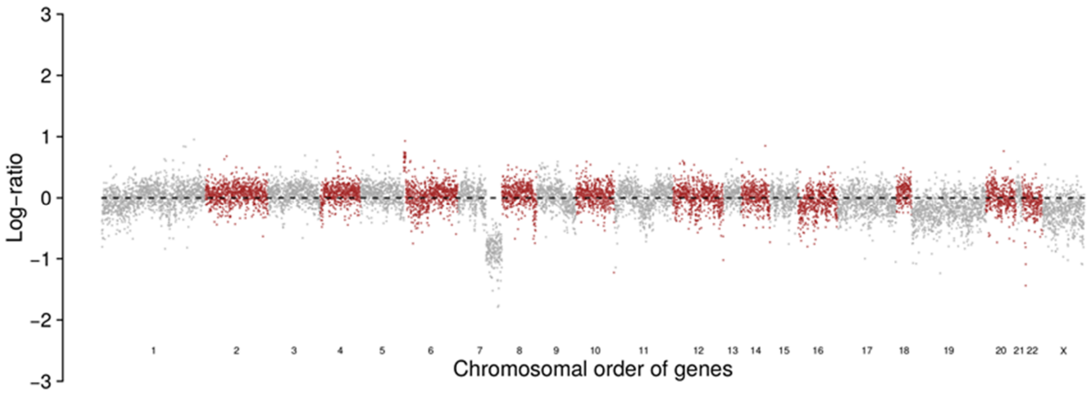

Case 36

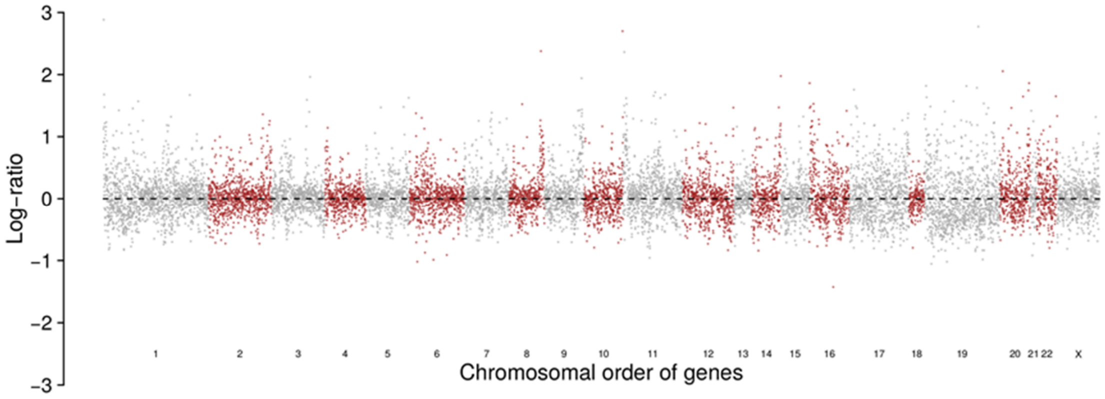

Case 101

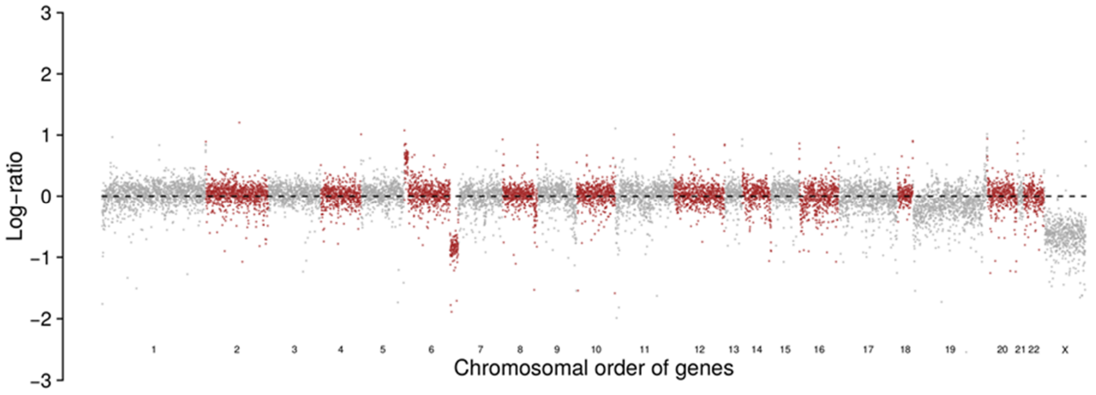

Case 102

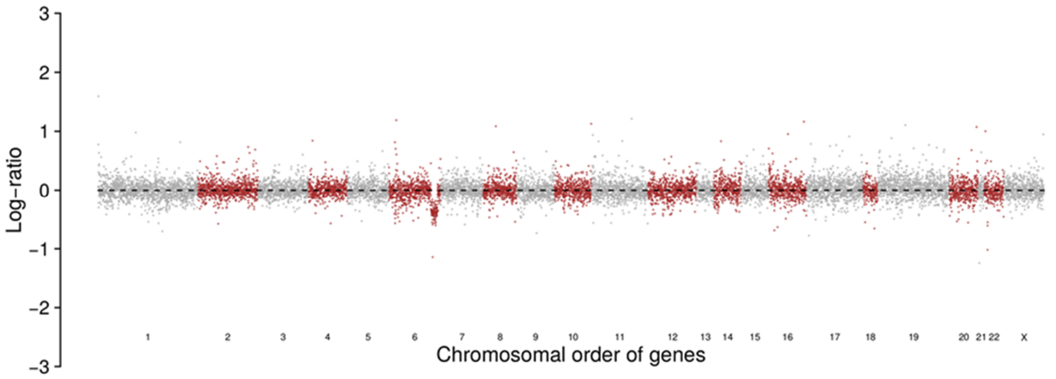

Figure S1. Gene copy number alteration profiles of six OAML. Gene copy number alterations are quantified by log2-ratios of tumor to normal based on WGS data. Log-ratios are plotted in the chromosomal order of genes from chromosome 1 to X for each individual tumor-normal pair. Chromosomes are additionally separated by alternating grey and brown dots. Strong deviations of log-ratios from zero indicate deletions (negative log-ratios) and duplications (positive log-ratios) of genes in tumor. Details are described in the material and methods section of the main manuscript.

Table S1. Overview of clinical data and the sequencing analyses performed.

OAL sample	Sex	Age at diagnosis	Localisation	WGS	WES	Targeted seq	Metagenomic analysis
1	f	67	conjunctiva	x		x	
2	m	70	conjunctiva			x	
3	m	76	eye lid		x	x	
4	m	62	conjunctiva			x	x
5	m	40	eye lid			x	
6	m	60	orbit	x		x	
7	f	40	lacrimal gland		x	x	
8	m	87	orbit			x	
9	f	64	lacrimal gland			x	
10	f	58	n.a.			x	
11	f	78	orbit			x	
12	m	54	eye lid			x	x
13	f	24	conjunctiva			x	
14	f	65	conjunctiva			x	
15	f	64	conjunctiva			x	
16	f	83	orbit			x	
17	f	64	orbit			x	
20	f	86	orbit			x	
21	m	75	orbit			x	
22	f	57	orbit			x	x
23	m	65	eye lid			x	
26	f	67	orbit			x	
27	f	62	conjunctiva			x	
28	f	29	conjunctiva			x	
30	m	82	n.a.			x	
31	m	60	conjunctiva	x		x	
32	m	76	eye lid			x	
34	f	62	eye lid			x	x
35	m	77	orbit			x	
36	f	34	conjunctiva	x		x	x
37	m	82	eye lid			x	
38	f	41	orbit			x	
40	f	60	eye lid		x	x	x
43	n.d.	n.d.	n.a.			x	
44	n.d.	n.d.	n.a.			x	
46	n.d.	n.d.	n.a.			x	
47	n.d.	n.d.	n.a.			x	
48	n.d.	n.d.	n.a.			x	
49	m	89	orbit			x	
51	m	76	orbit			x	x
52	m	78	eye lid			x	
53	m	87	n.a.			x	
54	m	79	orbit			x	
55	f	67	conjunctiva			x	
56	m	39	lacrimal gland			x	
57	f	75	conjunctiva			x	
59	f	65	eye lid			x	
60	f	54	n.a.			x	x
61	f	56	conjunctiva			x	
62	f	58	eye lid			x	
63	m	66	orbit			x	
64	m	80	conjunctiva			x	x
66	m	76	orbit			x	x
68	m	76	n.a.			x	
69	m	37	orbit		x	x	x
71	f	59	orbit			x	
72	m	62	conjunctiva			x	
73	f	75	conjunctiva			x	
74	m	76	n.a.			x	x
75	m	64	n.a.			x	
76	f	89	orbit		x	x	x
77	m	44	orbit			x	

78	m	69	eye lid			x	
79	f	45	orbit			x	
80	m	66	lacrimal gland			x	
81	f	69	eye lid			x	
82	m	63	eye lid			x	
83	m	78	lacrimal gland			x	
84	m	64	orbit			x	
85	m	78	lacrimal gland			x	
86	f	68	orbit			x	
87	f	92	orbit		x	x	x
88	f	59	n.a.			x	x
89	m	79	orbit			x	
90	f	76	orbit			x	
92	f	74	eye lid			x	x
93	f	53	eye lid		x	x	
95	f	83	orbit			x	
96	m	67	eye lid			x	
97	f	62	lacrimal gland			x	x
98	m	42	conjunctiva			x	
99	f	55	conjunctiva			x	
101	m	71	orbit	x	x		
102	f	41	orbit	x			

Table S2. Mutations identified in the 38 genes analyzed by targeted deep sequencing.

Cases	Approach	TBL1XR1	COL12A1	COL1A2	CXCR4	THBS3	RPS6KA5	LAMA3	ACTG1	CELSR1	EPHA1	ADAMTS13	CREBBP	DOCK8	IRF7	EHMT2	RIPK4	TBX1	PMS1	RYR1	TLR4	CDK12	ADAMTS5	APOBEC3B	ELF4	JAK1	JAK3	COL5A1	ETFB	GIGYF2	SCARB1	SCARF2	ACTN4	C1R	CHRNA3	CHRNB1	CHRNB4	KRT6B	OPRL1
1	WGS												1					1		1							1	1				2							
1	TGS																										1												
2	TGS																																						
3	WES		1																								1												
3	TGS																										1												
4	TGS																																						
5	TGS																																						
6	WGS											1				1														1	1					1	1		
6	TGS																																						
7	WES	1		1						1											1	1											1						
7	TGS	2								1											1	1			1								1						
8	TGS				1										1																								
9	TGS																																						
10	TGS																										1												
11	TGS																																						
12	TGS																						1																
13	TGS												1																									1	
14	TGS												1																									1	
15	TGS	1																																				1	
16	TGS	1																																					
17	TGS																						1																
20	TGS																			1																			
21	TGS		1																																				
22	TGS										1															1	1												
23	TGS																																						
26	TGS	1	2				1	1					1							1																			
27	TGS																			1																			
28	TGS																																						
30	TGS																																						
31	WGS	1	1						1			1						1					1						1		1	2						1	1
31	TGS	1	1																				2																
32	TGS																																						
34	TGS							1																				1											
35	TGS													1																									
36	WGS																																		1				
36	TGS	1																																					
37	TGS												1																										
38	TGS	1											1																										
40	WES						1																																
40	TGS																																						
43	TGS			1										1																									
44	TGS																																						
46	TGS																																						

**47**	**TGS**																							
**48**	**TGS**																							
**49**	**TGS**							1			1									1				
**51**	**TGS**																							
**52**	**TGS**																							
**53**	**TGS**																							
**54**	**TGS**											1	1											
**55**	**TGS**										1													
**56**	**TGS**											1												
**57**	**TGS**																							
**59**	**TGS**							1																
**60**	**TGS**																							
**61**	**TGS**										1								1					
**62**	**TGS**																							
**63**	**TGS**																							
**64**	**TGS**													1										
**66**	**TGS**																							
**68**	**TGS**																							
**69**	**WES**					1		1		1														
**69**	**TGS**		1			1				1														
**71**	**TGS**									1														
**72**	**TGS**												1				1					1		
**73**	**TGS**	1																1						
**74**	**TGS**																						1	
**75**	**TGS**											1												
**76**	**WES**			1			1																	
**76**	**TGS**			1																				
**77**	**TGS**			1												1								
**78**	**TGS**																							
**79**	**TGS**				1																			
**80**	**TGS**	1																					1	
**81**	**TGS**								1				1											
**82**	**TGS**																							
**83**	**TGS**		1						1										1					
**84**	**TGS**																							
**85**	**TGS**	1					1																	
**86**	**TGS**		3	1				1	3		2	2	1						1			1		
**87**	**WES**	1									1													
**87**	**TGS**	1													1				1					
**88**	**TGS**	1																			1			
**89**	**TGS**																							1
**90**	**TGS**																							
**92**	**TGS**											1												
**93**	**WES**																			1				
**93**	**TGS**																							
**95**	**TGS**																							
**96**	**TGS**			1																				
**97**	**TGS**																							

**98**	**TGS**						2												1			
**99**	**TGS**																					
**101**	**WGS**	1	1			1		1	1	1	1	2						1				1
**101**	**WES**		1	1	1																	
**102**	**WGS**												1	1	1	1	1			1	2	1

Table S4. Significant recurrent enrichment of gains and losses in six OAML studied by WGS.

Chromo-some	Start[1]	Stop[1]	Genes	Number of cases	kind	p value[2]	Lfdr[3]
1	895968	901095	*KLHL17*	4	gain	4.23E-05	0.0017
1	934343	935552	*HES4*	4	gain	4.23E-05	0.0017
1	41326730	41328018	*CITED4*	4	gain	4.23E-05	0.0017
1	47881745	47883723	*FOXE3*	4	gain	4.23E-05	0.0017
1	63788731	63790797	*FOXD3*	4	gain	4.23E-05	0.0017
1	200842084	200843306	*GPR25*	4	gain	4.23E-05	0.0017
2	176964459	176966408	*HOXD12*	4	gain	4.23E-05	0.0017
2	219824378	219826876	*CDK5R2*	4	gain	4.23E-05	0.0017
3	137483580	137484396	*SOX14*	4	gain	4.23E-05	0.0017
6	138188352	138204449	*TNFAIP3*	3	loss	1.90E-07	0.0004
7	122634760	122635754	*TAS2R16*	2	loss	6.76E-05	0.0029
8	48649472	48651648	*CEBPD*	4	gain	4.23E-05	0.0017
8	144239332	144242128	*LY6H*	4	gain	4.23E-05	0.0017
9	131937836	131940540	*IER5L*	4	gain	4.23E-05	0.0017
11	626432	627143	*SCT*	4	gain	4.23E-05	0.0017
11	2289726	2292182	*ASCL2*	4	gain	4.23E-05	0.0017
11	17741116	17743678	*MYOD1*	4	gain	4.23E-05	0.0017
11	64008476	64011604	*FKBP2*	4	gain	4.23E-05	0.0017
11	82443054	82444906	*FAM181B*	4	gain	4.23E-05	0.0017
12	49687036	49692465	*PRPH*	4	gain	4.23E-05	0.0017
13	95361887	95364389	*SOX21*	4	gain	4.23E-05	0.0017
14	95234554	95236562	*GSC*	4	gain	4.23E-05	0.0017
16	230453	231180	*HBQ1*	4	gain	4.23E-05	0.0017
16	2285818	2288712	*DNASE1L2*	4	gain	4.23E-05	0.0017
16	86600858	86602539	*FOXC2*	4	gain	4.23E-05	0.0017
17	4692255	4693685	*GLTPD2*	4	gain	4.23E-05	0.0017
17	37760022	37764196	*NEUROD2*	4	gain	4.23E-05	0.0017
17	59489113	59490641	*C17orf82*	4	gain	4.23E-05	0.0017
17	80332154	80333462	*UTS2R*	2	loss	6.76E-05	0.0029
19	859666	863606	*CFD*	4	gain	4.23E-05	0.0017
19	859666	863606	*CFD*	2	loss	6.76E-05	0.0029
19	917343	921014	*KISS1R*	4	gain	4.23E-05	0.0017
19	1103937	1106786	*GPX4*	4	gain	4.23E-05	0.0017
19	14583279	14586174	*PTGER1*	4	gain	4.23E-05	0.0017
19	18390564	18392432	*JUND*	4	gain	4.23E-05	0.0017
19	18390564	18392432	*JUND*	2	loss	6.76E-05	0.0029
19	47137334	47137939	*GNG8*	4	gain	4.23E-05	0.0017
19	51226606	51228979	*CLEC11A*	4	gain	4.23E-05	0.0017
19	55888205	55889612	*TMEM190*	4	gain	4.23E-05	0.0017
20	48807377	48809212	*CEBPB*	4	gain	4.23E-05	0.0017
20	61637332	61638387	*BHLHE23*	4	gain	4.23E-05	0.0017
21	34442451	34444726	*OLIG1*	4	gain	4.23E-05	0.0017
X	102470021	102472174	*BEX4*	2	loss	6.76E-05	0.0029
X	153237779	153248646	*TMEM187*	2	loss	6.76E-05	0.0029
X	153880247	153881853	*CTAG2*	4	gain	4.23E-05	0.0017

© 2020 by the authors. Submitted for possible open access publication under the terms and conditions of the Creative Commons Attribution (CC BY) license (http://creativecommons.org/licenses/by/4.0/).
